# Prevalence of burnout among health care workers in the Federation of Bosnia and Herzegovina during the coronavirus disease-2019 pandemic: a cross-sectional study

**DOI:** 10.3325/cmj.2022.63.482

**Published:** 2022-10

**Authors:** Ankica Mijić Marić, Marnela Palameta, Amra Zalihić, Marija Bender, Mirela Mabić, Marina Berberović, Sandra Kostić

**Affiliations:** 1 Department of Family Medicine, Health Care Center Mostar, School of Medicine University of Mostar, Mostar, Bosnia and Herzegovina; 2 Department of Neurology, School of Medicine University of Mostar, Mostar, Bosnia and Herzegovina; 3 Faculty of Economics, University of Mostar, Mostar, Bosnia and Herzegovina; 4 University Clinical Hospital Mostar, Emergency Medicine Center, Mostar, Bosnia and Herzegovina; 5 Department of Anatomy, Histology and Embryology, University of Split School of Medicine, Split, Croatia

## Abstract

**Aim:**

To investigate the prevalence of burnout syndrome among health care workers in the Federation of Bosnia and Herzegovina (FBiH) during the coronavirus disease 2019 (COVID-19) pandemic.

**Methods:**

This cross-sectional study was conducted in May and June 2021 using an online survey based on Copenhagen Burnout Inventory. The questionnaire underwent forward and backward translation, preliminary pilot testing, and was assessed for reliability and validity. Personal burnout, work-related burnout, and patient-related burnout were assessed. The survey was sent to the members of the Union of Physicians and Dentists in FBIH, who were asked to forward the link to their medical technicians and nurses.

**Results:**

A total of 77% of participants experienced some form of burnout. As many as 32% experienced all three forms of burnout. Those actively involved in tackling the COVID-19 pandemic more often experienced burnout. In personal and work-related burnout domains, higher level of burnout was reported among female respondents. Higher work-related and patient-related burnout was reported by physicians compared with medical technicians/nurses. Higher level of patient-related burnout was reported in health care workers aged 30-39 and 50-59 years, among respondents working in primary care, and among physicians.

**Conclusion:**

The majority of health care workers showed moderate or high levels of personal and work-related burnout, with a lower level of patient-related burnout. There is a need for further research into the causes of burnout, as well as for the implementation of organizational interventions aimed to minimize workplace burnout.

Burnout syndrome (BOS) was added to the 11th edition of the International Classification of Diseases (ICD-11) by the World Health Organization as “a syndrome resulting from chronic workplace stress that has not been successfully managed” (1). BOS is a frequent public-health and workplace issue, with rising prevalence and incidence (2). This issue has particularly came into focus during the coronavirus disease 2019 (COVID-19) outbreak. It can happen at any age, as well as during residency (3), and result in medical errors and poor patient care (4).

BOS has a variety of causes and predictions, both environmental and personal, which are not all related to workload. Administration, intrinsic factors of work, contact with patients, financial stressors, interference of work and social life, organizational structure and atmosphere, and relationships with coworkers, are only some of them (5,6).

Burnout among health care workers (HCWs) was frequent even before the COVID-19 pandemic, and it is a significant health concern for the global economy because of its effects on organizational and patient outcomes (7). BOS has also been linked to depression, anxiety, and posttraumatic stress disorder in HCWs, as well as to lower satisfaction and care quality, and a higher suicide rate (8,9).

The COVID-19 pandemic has put HCWs under a lot of stress (10). Burnout among HCWs is a significant problem since it affects not only HCWs themselves, but also their families, patients, and society. This is why it is necessary to create strategies for dealing with burnout (11). Burnout has been identified as one of the threats to the stability of health care professionals in the fight against COVID-19 (12-15).

Acute stress is more likely to cause sleep disturbances, anxiety, fear, mood changes, as well as posttraumatic stress disorder. Chronic stress, on the other hand, is more likely to cause BOS, which is a condition closely connected to poor job ability (16,17).

Job burnout can put the individual’s well-being and health at risk, but it can also result in medical errors and poor patient care (4). As a result, it is important to identify factors that lead to job-related burnout and stress among HCWs. This knowledge may be used to protect the workers while improving the quality of services provided to the patients (18).

The aim of our study was to assess the prevalence of burnout syndrome among health care workers in Federation of Bosnia and Herzegovina (FBiH), one of the two entities within Bosnia and Herzegovina, during the COVID-19 pandemic, as this issue has not been addressed so far.

## Material and methods

### Study design

This cross-sectional study was carried out between May 26 and July 26, 2021 with the use of an online survey based on Copenhagen Burnout Inventory (CBI). Google Forms platform was used to collect data. An invitation to participate in the online survey was sent to the members of the Union of Physicians and Dentists in FBiH, who were asked to forward the link to their medical technicians and nurses (snowball sampling technique). The consent to use the communication channels of the Union was obtained previously (number: 01-8/2021, date: May 4, 2021). The online survey also included an introductory note stating the study aim. Research participation was voluntary and anonymous. Inclusion criteria were set to include health care workers in the FBiH, regardless of their age, health care status, job title, or employment status. Request to participate was resent again after one month, and data collection ended on July 26. The study was approved by the Committee for Medical Ethics of the Faculty of Medicine University of Mostar (01-I-854/21).

### Instrument/measurement

The first part of the questionnaire inquired about gender, age, children, job profile, work environment, and involvement in detection, surveillance, and treatment of patients with COVID-19.

The second part, the CBI, consists of 19 specific questions on burnout divided into three sections. The first section consists of six items on personal burnout, ie, general symptoms of exhaustion. The second section consists of seven items on work-related burnout, ie, symptoms of exhaustion associated to work. The third subscale consists of six items on client-related burnout, ie, symptoms of exhaustion associated with working with clients. The inventory was adapted by replacing the word “client” with the word “patient” given our target population. Twelve items of CBIs assess frequency (response categories: always, often, sometimes, seldom, never/almost never) and seven items assess intensity (response categories: to a very high degree, to a high degree, somewhat, to a low degree, to a very low degree). Scoring was performed as follows: always/to a very high degree: 100; often/to a high degree: 75; sometimes/somewhat: 50; seldom/to a low degree: 25; never/almost never /to a very low degree: 0. According to the questionnaire instructions, reverse scoring was performed for one item/question. Total score on the scale and subscale is the average of the scores on the corresponding items/questions. Burnout was defined as CBI score >50, higher score represents a higher level of burnout (score 50 to 74 – moderate, 75 to 99 – high, and 100 – severe burnout) (19-22).

### Validity and reliability

We conducted forward and backward translation and preliminary pilot testing of the questionnaire. The Kaiser-Meyer-Olkin measure of the sampling adequacy was 0.954, and Bartlett’s test of sphericity was significant (*P* < 0.001). Three factors explained 68.32% of the variance: factor 1 explained 54.01%, factor 2 explained 9.57%, and factor 3 explained 4.74% of the variance. The factor loadings varied from 0.541 to 0.850. The CFA confirmed the distribution of items from the questionnaire according to the defined dimensions. The Cronbach’s alpha for the whole scale was 0.936, and the interclass correlation was 0.954. The values of the Cronbach’s alpha coefficient for individual subscales were as follows: personal burnout scale 0.895, work-related burnout scale 0.909, and patient-related burnout scale 0.909.

### Respondents

During the period when the survey was available 849 participants completed the questionnaire. Nine responses were excluded since those respondents were not HCWs. Finally, 840 responses were included in the analysis, out of which 520 were from the Union of Physicians and Dentists, and 320 were medical technicians/nurses and other HCWs. In 2019, the Union of Physicians and Dentists in FBIH had 4321 members (23), which is over 90% of the total number in FBiH (24). With these data, we calculated the response rate for physicians and dentists, which was 12% (520/4312). The sampling method (snowball sampling technique) prevented us from calculating the response rate for medical technicians/nurses and other HCWs.

Most of the respondents were women, were aged between 30 and 50 years, had up to 20 years of service, were physicians, had a college degree, were married, had children, and worked in primary care (Table 1). HCWs from each of the ten counties in FBiH took part in the survey. Among physicians, there were 291 (59.6%) specialists, 102 (20.9%) trainees, and 95 (19.5%) graduate physicians without specialization.

**Table 1:** Respondents’ characteristics

	Number of respondents	% (n = 840)
Sex		
male	200	23.8
female	640	76.2
Age (year)		
≤30	141	16.8
31-39	293	34.9
40-49	212	25.2
50-59	133	15.8
60+	61	7.3
Number of children		
no	293	34.9
1	168	20.0
2	274	32.6
>2	105	12.5
Work experience (years)		
0-10	337	40.1
11-20	270	32.1
21-30	132	15.7
30+	101	12.0
Education		
high school	185	22.0
college	532	63.3
MSc or PhD	123	14.6
Level of health care		
primary care	483	57.5
secondary care	178	21.2
tertiary care	150	17.9
other	29	3.5
Qualification		
physicians	488	58.1
dentists	32	3.8
medical technicians/nurses	267	31.8
other HCWs	53	6.3

### Statistical analysis

The normality of the distribution of quantitative data was assessed with the Kolmogorov-Smirnov test. Data are presented as mean and standard deviation (SD). Differences between groups were tested with a *t* test for independent samples and one-way ANOVA. For categorical data, frequencies and percentages were calculated. The validity of the used instrument was verified by confirmatory factor analysis (CFA). The internal consistency/reliability was checked with Cronbach’s alpha coefficient (CA) and interclass correlation. The significance limit was set at *P* = 0.05. Data were analyzed with IBM SPSS for Windows, version 25.0 (IBM Corp., Armonk, NY, USA).

## RESULTS

The mean total burnout score was 49.5 ± 17.8. The mean score per CBI scale was57.23 ± 17.13 for personal burnout, 49.68 ± 20.80 for work-related burnout, and 41.43 ± 21.12 for patient-related burnout. According to the results for total burnout, no respondent had a score of 100, while almost half of the respondents showed the symptoms of burnout (49.1%; n = 413).

Differences in burnout were observed according to age, active involvement in detection and treatment of COVID-19 patients, and qualification. Among respondents with burnout, there were more physicians, and HCWs aged 30-39 involved in the detection, follow-up, and treatment of COVID-19 patients (Table 2).

**Table 2:** Demographic characteristics of respondents with burnout vs without burnout

	Number (%) of respondents		
	with burnout (n = 413)	without burnout (n = 427)	P*
Sex			0.177
male	90 (21.8)	110 (25.8)	
female	323 (78.2)	317 (74.2)	
Age (year)			0.009
≤30	62 (15.0)	79 (18.5)	
31-39	163 (39.5)	130 (30.4)	
40-49	89 (21.5)	123 (28.8)	
50-59	73 (17.7)	60 (14.1)	
60+	26 (6.3)	35 (8.2)	
Work experience (years)			0.144
0-10	158 (38.3)	179 (41.9)	
11-20	146 (35.4)	124 (29.0)	
21-30	57 (13.8)	75 (17.6)	
30+	52 (12.6)	49 (11.5)	
Active in detection and treatment of COVID-19 patients			
yes	377 (91.3)	313 (73.3)	<0.001
no	36 (8.7)	114 (26.7)	
Level of health care			
primary care	245 (61.9)	238 (57.3)	0.190
secondary care	151 (38.1)	177 (42.7)	
Qualification			
physicians	257 (68.4)	231 (60.9)	0.033
medical technicians/nurses	119 (31.6)	148 (39.1)	
Specialization			
no specialization	54 (21.0)	41 (17.7)	0.613
trainees	51 (19.8)	51 (22.1)	
specialists	152 (59.1)	139 (60.2)	

*χ^2^ test.

Respondents showed a higher level of personal and work-related burnout compared with the level of patient-related burnout. A total of 72.9% of respondents (n = 612) had moderate or high score on the personal burnout domain, 50.9% of respondents (n = 428) experienced moderate or high work-related burnout, and 38.3% respondents (n = 322) reported a lower level of patient-related burnout (Table 3).

**Table 3:** Level of burnout, in general and by dimensions

	Number (%) of respondents with			
Burnout	personal burnout	work related burnout	patient related burnout	total burnout
No	228 (27.1)	412 (49.0)	518 (61.7)	427 (50.8)
Moderate	449 (53.5)	300 (35.7)	263 (31.3)	348 (41.4)
High	157 (18.7)	126 (15.0)	53 (6.3)	65 (7.7)
Severe	6 (0.7)	2 (0.2)	6 (0.7)	0

Overall, 77% of participants experienced some form of burnout (in at least one dimension of burnout they had a score higher than 50%). A total of 24.2% of participants experienced only one form of burnout, 20.6% two forms of burnout, and 32.3% participants experienced all three forms of burnout.

More than 80% of participants were involved in the detection, follow-up, and treatment of COVID-19 patients (n = 690). Over half of the respondents reported that they had had COVID-19 infection (54%, n = 459), 61% (n = 516) had been vaccinated against SARS-CoV-2 virus, while 16% (n = 134) had been neither vaccinated nor had had COVID-19 infection.

Out of 745 respondents who answered to the question: “If you feel you have burnout professionally, do you think it started before or after the onset of the COVID-19 pandemic?”, 36% (n = 268) answered that they experienced it before, and 64% (n = 477) answered that they experienced it after the onset of the pandemic.

Female respondents reported a higher burnout level in the personal and work-related burnout domains. Respondents who were involved in detection, follow-up, and treatment of COVID-19 patients had a higher burnout incidence in all domains. Higher work-related burnout was reported by physicians. Higher patient-related burnout was reported by HCWs aged 30-39 and 50-59 years, by respondents working in primary care, and by physicians (Table 4).

**Table 4:** Dimensions of burnout in regard to the respondents’ characteristics

	Mean (standard deviation)		
	personal burnout	work-related burnout	patient-related burnout
Sex			
male	51.63 (18.48)	46.50 (21.88)	41.89 (22.20)
female	58.98 (16.59)	50.67 (20.37)	41.28 (20.78)
P*	<0.001	0.017	0.729
Age (year)			
≤30 (A)	56.42 (15.44)	49.09 (20.92)	39.36 (19.14)
31-39 (B)	58.08 (16.74)	51.35 (20.28)	43.91 (21.06)
40-49 (C)	56.99 (18.12)	47.39 (20.26)	36.26 (20.69)
50-59 (D)	58.05 (17.54)	51.13 (21.29)	46.39 (21.02)
60+ (E)	53.96 (20.79)	47.78 (23.48)	41.39 (23.58)
P^†^	0.482	0.220	<0.001 (C-B,D)
Work experience (years)			
0-10	56.58 (17.09)	49.12 (20.73)	40.50 (20.19)
11-20	58.35 (17.39)	51.20 (20.18)	42.45 (21.45)
21-30	57.26 (16.09)	47.97(20.99)	40.03 (21.52)
30+	56.39 (19.52)	49.68 (22.42)	43.61 (22.69)
P^†^	0.607	0.461	0.402
Active in detection, follow-up, and treatment of COVID-19 patients			
yes	58.94 (16.61)	52.21 (20.27)	43.35 (21.04)
no	49.39 (18.44)	38.02 (19.26)	32.61 (19.23)
P*	<0.001	<0.001	<0.001
Level of health care			
primary care	57.58 (16.79)	49.91 (20.56)	44.00 (21.11)
secondary care	56.22 (18.10)	48.94 (21.08)	37.20 (20.40)
P*	0.274	0.515	<0.001
Qualification			
physicians	57.90 (17.38)	51.44 (20.93)	43.09 (21.43)
medical technicians/nurses	56.16 (17.20)	47.57 (20.28)	38.29 (20.04)
P*	0.192	0.016	0.003
Specialization			
no specialization(A)	59.61 (16.55)	52.97 (20.79)	47.15 (22.66)
trainees (B)	57.03 (17.17)	49.72 (20.89)	37.30 (19.33)
specialists (C)	57.56 (17.67)	51.34 (21.07)	43.67 (21.16)
P^†^	0.527	0.554	0.004 (B-A,C)

*t test for independent samples.

†one-way ANOVA.

## DISCUSSION

The CBI questionnaire revealed that 77% of participants experienced some form of burnout, and as many as 32% experienced all three forms of burnout. This leads to the conclusion that burnout is a frequent issue among HCWs in FBiH.

The majority of HCWs showed moderate or high levels of personal and work-related burnout, and lower levels of patient-related burnout. Similar results were obtained among Portuguese HCWs working at the time of the COVID-19 pandemic, with a high level of personal (52.5%) and work-related burnout (53.1%), and a lower level of patient-related burnout (35.4%) (25). Before the pandemic, lower scores of patient-related burnout were observed compared with other domains (26,27). Burnout was mainly related to increased quantitative workload, increased job insecurity, and lower job satisfaction (26).

In a meta-analysis by Batra et al (28), the overall prevalence of burnout among HCWs was 37.4%. During SARS and MERS outbreaks, BOS was experienced by approximately one-third of HCWs (29). Although the connection between BOS and COVID-19 is still being investigated, the prevalence rates are similar to those in prior epidemics (29). The pooled burnout prevalence in HCWs during SARS/MERS/SARS-CoV-2 epidemics was 34.4% (30). As most of the mentioned research assessed burnout with the Maslach Burnout Inventory (MBI), a comparison of these prevalence rates with our results should be made with caution.

In our study, female respondents reported a higher burnout level in the personal and work-related burnout domains. Although this may be explained by the majority of the respondents being women, these findings are consistent with earlier reports (11,31). Higher work-related burnout was reported by physicians. In the patient-related burnout domain, higher level of burnout was reported by HCWs aged 30-39 and 50-59 years, by respondents working in primary care, and by physicians compared with medical technicians/nurses.

Respondents who were involved in the detection, follow-up, and treatment of COVID-19 patients had a higher burnout prevalence in all domains. Over 64% of respondents considered that their burnout started after the onset of the pandemic. A high prevalence of pandemic-related burnout was reported in India (32). In a rapid global survey conducted during the COVID-19 pandemic, half of the respondents from 33 countries experienced burnout as a result of their work (31). Physicians in primary care in Portugal experienced a higher rate of burnout in comparison with pre-COVID-19 levels (33).

According to the research on mental health disorders (MHD) among family physicians in Croatia during the COVID-19 pandemic, respondents with positive MHD history had lower scores on resilience, healthy lifestyle, and satisfaction with life and work, but higher scores on burnout (34). Nearly 50% of MHD positive physicians developed these disturbances in the COVID-19 pandemic era (34). These results support the idea of a healthy lifestyle and resilience as key protective factors against work-related burnout (34).

A study from China reported a higher frequency of insomnia, depression, anxiety, and mental distress among HCWs actively engaged in the detection, diagnosing, and treatment of COVID-19 patients (35).

Burnout among BiH physicians was investigated before the pandemic among physicians in primary health care from Banja Luka. The study was based on the MBI, and 20.9% of respondents had a high level of emotional exhaustion, 43.2% had a high level of depersonalization (36), and emotional exhaustion and depersonalization were suggested to be the foundation of burnout (5). Another survey based on MBI, conducted among primary health care providers in the Sarajevo Canton, showed that 25.3% of HCWs experienced a high level of emotional exhaustion and 17.7% experienced depersonalization (37). Research conducted among physicians in Croatia on a national level before the pandemic showed that 63% of all physicians experienced burnout, and 16% of physicians experienced all three forms of burnout (38). The research on the levels of burnout syndrome in critical care nurses in Croatia showed that 22.1% of nurses expressed a high level of emotional exhaustion, with a lower level of depersonalization (7.9%), and 34.5% had a low level of personal accomplishment (39).

There are several potential limitations to this study. Our sample may not entirely represent all the HCWs in FBiH, and may be biased toward HCWs whose working circumstances are unsatisfactory. Those who did not experience burnout may have opted not to respond. Furthermore, we did not investigate the causes or effects of burnout.

In conclusion, burnout is worryingly common in the population of HCWs in FBiH. Further research into the causes of burnout is needed. Active measures are required to minimize high burnout prevalence among HCWs in FBiH.
